# School lunch and snacking patterns among high school students: Associations with school food environment and policies

**DOI:** 10.1186/1479-5868-2-14

**Published:** 2005-10-06

**Authors:** Dianne Neumark-Sztainer, Simone A French, Peter J Hannan, Mary Story, Jayne A Fulkerson

**Affiliations:** 1 Division of Epidemiology and Community Health, University of Minnesota, Minneapolis, Minnesota, USA; 2 School of Nursing, University of Minnesota, Minneapolis, Minnesota, USA

## Abstract

**Objectives:**

This study examined associations between high school students' lunch patterns and vending machine purchases and the school food environment and policies.

**Methods:**

A randomly selected sample of 1088 high school students from 20 schools completed surveys about their lunch practices and vending machine purchases. School food policies were assessed by principal and food director surveys. The number of vending machines and their hours of operation were assessed by trained research staff.

**Results:**

Students at schools with open campus policies during lunchtime were significantly more likely to eat lunch at a fast food restaurant than students at schools with closed campus policies (0.7 days/week vs. 0.2 days/week, p < .001). Student snack food purchases at school were significantly associated with the number of snack machines at schools (p < .001) and policies about the types of food that can be sold. In schools with policies, students reported making snack food purchases an average of 0.5 ± 1.1 days/week as compared to an average of 0.9 ± 1.3 days/week in schools without policies (p < .001). In schools in which soft drink machines were turned off during lunch time, students purchased soft drinks from vending machines 1.4 ± 1.6 days/week as compared to 1.9 ± 1.8 days/week in schools in which soft drink machines were turned on during lunch (p = .040).

**Conclusion:**

School food policies that decrease access to foods high in fats and sugars are associated with less frequent purchase of these items in school among high school students. Schools should examine their food-related policies and decrease access to foods that are low in nutrients and high in fats and sugars.

## Background

Research studies have clearly shown that adolescents' dietary intakes are not consistent with national recommendations. Areas of concern include high intakes of saturated fat, total fat, and soft drinks, and low intakes of fruits, vegetables, fiber, and calcium-rich foods [1-3]. These dietary patterns are of concern because of their potential for increasing risk for developing obesity, heart disease, osteoporosis, dental caries, and various types of cancer [4].

Adolescent eating patterns are influenced by factors proximal to the adolescent such as individual food preferences [5], family meal patterns [6], and parental role modeling [7]. However, it is increasingly becoming clearer that adolescent eating patterns are also influenced by more distal factors such as media messages and social norms [8,9]. Since adolescents spend a large amount of time in school, an important question – is to what extent does the school food environment influence adolescent eating patterns [10]. In Bronfenbrenner's ecological model, which shows concentric spheres of influences on the individual ranging from proximal factors (i.e., individual characteristics) to distal factors (i.e. social norms and public policies), the school lies in the middle [11].

Although there are federal regulations regarding the types of foods that can be served in the United States Department of Agriculture (USDA) reimbursable school meals [12,13], few regulations are in place for alternative foods, such as those served a la carte in the cafeteria or in snack bars, and in vending machines [14]. A study of 55 high schools revealed that school environments do not always foster healthful eating practices consistent with national dietary guidelines [15]. In a statewide survey of food policies in 336 Minnesota high schools, two-thirds of the principals indicated that only healthy food choices should be provided to students at school, yet only one-third reported that their school had an overall policy about nutrition and food. Even fewer reported the presence of specific policies about the types of foods and beverages sold in vending machines, school stores, or at school functions [16]. School vending machines were prevalent and 77% of the principals reported that their school or district had a contract with a soft drink company. Vending machine hours were limited in some way in 81% of the schools, but only about a third of the schools limited the vending machine hours to before and after school only or after all lunch periods were completed. While it is important to respect adolescents increasing autonomy and decision-making skills, research clearly shows that food availability is one of the strongest correlates of food choices in adolescents [17,18]. Schools provide a setting in which it is possible to increase the availability and attractiveness of a range of healthy food options from which students can make choices, and restrict the availability of foods that are low in nutrients and high in fats and sugars.

The few studies that have examined associations between school food environment and student eating patterns suggest that the school food environment has a significant impact on food choices. Cullen and colleagues [19] found that fourth grade students attending elementary schools without a la carte food items consumed more fruits, juices, and vegetables than fifth graders who attended a middle school with a la carte food line (or snack bar). Kubik et al found that among seventh-grade students in 16 schools, having a school a la carte program was associated with lower intakes of fruits and vegetables and higher intakes of calories from total and saturated fats [20]. They also found that the number of snack vending machines was associated with lower fruit intakes, suggesting that students may be choosing alternative snack foods from the vending machines rather than fruit. However, policies were not examined regarding the types of foods sold in vending machines and their hours of operation and the impact of such policies on vending machine purchases.

This study expands on the limited, but growing, body of literature that explores the role of schools in influencing the dietary practices of youth. Specifically, our study objectives are: 1) to describe school lunch practices and vending machine purchases in a large sample of high school students; and 2) to examine associations between eating patterns of high school students and school food environment and policies.

## Methods

### Study population and study design

The study population included 1088 high school students from 20 high schools in the Minneapolis/St. Paul metropolitan area in Minnesota. These schools were participating in TACOS (Trying Alternative Cafeteria Options in Schools), a two-year, group-randomized, school-based nutrition intervention trial [21,22]. Participating schools were predominantly suburban, and ranged in enrollment from 812–3157 students (median = 1713). The study population was nearly equally divided on gender (47.0% male, 53.0% female). All students were in 9^th^–12^th ^grade (18.0%, 26.2%, 30.0%, and 25.8% in 9^th^, 10^th^, 11^th^, and 12^th ^grade, respectively). Race/ethnicity breakdown was as follows: 84.3% White, 4.6% Asian American, 2.5% Hispanic, 2.4% Black, and 6.2% American Indian/other). Nine percent of the students were eligible for free or reduced school lunch.

Data on adolescent school lunch patterns and vending machine practices were collected at baseline, prior to the beginning of the first year of the TACOS intervention with surveys that were mailed to the homes of a random sample of 75 students from each of the 20 participating schools. The University of Minnesota's Institutional Review Board Human Subjects Committee and the research review committees of the participating school districts approved all study protocols. A parental passive consent letter was included with the survey, as part of the cover letter; if the parent agreed to have their child participate they were asked to give the survey to their child. Students received ten dollars for completing the survey. The response rate for survey completion was 75%. Data on school food policies were collected with surveys that were mailed to principals and food service directors at each of the 20 participating schools at the end of the first intervention year. In one school, neither principal nor food service director responded, while in another school the principal did not respond but the food service director did, resulting in data from 19 schools from either the principal or the food service director. Questions on these surveys assessed school food-related policies and practices during the previous school year. The survey instrument was developed, based on previously published surveys about the school food environment [14-16,23-25]. Data on vending machine availability and hours of operation were collected – through site visits by trained research staff. Vending machines were included if they were in locations that were accessible to students (e.g., lunchroom, hallways, student locker areas, gymnasiums, commons area, etc., but not in faculty lounge areas).

### Description of measures

#### Adolescent Eating Patterns

*School lunch patterns *among adolescents were assessed with the following questions on the student survey: "During a normal school week, how may days per week do you...1) Get lunch in the school cafeteria main lunch line? 2) Get lunch in one of the school cafeteria a la carte or snack bar lines? 3) Bring lunch from home? 4) Get lunch off campus at a fast food restaurant? 5) Get lunch off campus at a convenience store?" *Vending machine practices *were assessed with two similar questions: "During a normal school week, how many days per week do you...1) Get food from a school snack/food vending machine?; and 2) Get soft drinks from a school vending machine?" Response categories were days per week (range: 0–5).

### School food-related policies and food environment

School food-related policies about open/closed campus during lunchtime and the types of food served in vending machines were assessed with the principal survey [22]. Data from the food service director survey were used in the analysis in the few instances in which there were missing data from the principal, or the principal gave the response: "don't know." The existence of a *closed campus policy during lunchtime *was assessed with the question: "Is the high school campus open or closed for lunch periods?" The existence of *policies about type of food sold in vending machines *was assessed with the question: "Are there any school policies about what is sold in the school vending machines (yes/no/don't know)?"

*Policies regarding hours of operation of vending machines during lunchtime *were determined for snack and soft drink vending machines through direct observations by research staff [22]. Direct observations by research staff were also used to assess the *number of vending machines *including number of snack vending machines, number of soft drink vending machines, and number of other vending machines. Snack vending machines were defined as those that were non-refrigerated, and sold items including candy bars, candy, chips, pretzels, pastry, and gum. Soft drink machines were defined as those that sold primarily soft drinks, but could include one or more sleeves of water, juice, juice drinks, or sports drinks. However, if more than half of the machine columns were filled with drinks other than soft drinks, the machine was recorded under "other" vending machines (e.g., fruit juice, juice drinks, water, or sports drinks).

### Data analysis

School food-related policies and measures of school environment (e.g., the number of vending machines) are school-level data implying that all students in a school are under the same policy. Adolescent eating patterns are individual-level data. In descriptive analyses of student eating patterns we present individual level means and standard deviations. For analyses of the association of school policies and student eating patterns, we used mixed models (SAS Release 8.2, proc MIXED) specifying the school as nested in the policy, that is, each student in a school is under the same policy. Standard errors for differences between categories of a policy are inflated, degrees of freedom are two fewer than the number of schools for which policy data is available.

## Results

### School lunch patterns and vending machine practices among students

Students more frequently ate meals from the main lunch line (Mean = 2.4 days/week) than any of the other options, although they also ate frequently from the a la carte line (M = 1.8 days/week) (Table 1). On average, students brought lunch from home once a week (M = 0.9 days/week). Lunch purchases from off-campus fast food restaurants and convenience stores were less frequent, although standard deviations were large, indicating variation across students. On average, students purchased snacks from vending machines nearly once a week (M = 0.9 days/week). Students purchased soft drinks from vending machines 1.6 days/week; nearly two-thirds (61.5%) of the students reported purchasing soft drinks at least one day/week.

**Table 1:** Lunch patterns andvending machine practices at school or during school hours in past school week (five days)

	Total N^†^	Mean days/week		Days/week (percentage reporting)			
		Mean	SD	0	1–2	3–4	5

**Lunch patterns:**							
Regular school lunch	1080	2.4	1.9	29.2	21.8	27.3	21.7
A la carte lunch	1077	1.8	1.7	31.6	36.2	22.0	10.2
Bring lunch	1076	0.9	1.7	71.9	9.8	7.5	10.8
Fast food restaurant	1075	0.4	1.0	81.6	13.0	3.8	1.6
Convenience store	1067	0.1	0.6	92.5	5.5	1.5	0.5
**Vending machine practices**:							
Vending snacks	1081	0.9	1.3	56.8	31.2	9.2	2.8
Vending soft drinks	1081	1.6	1.7	38.5	34.0	17.5	10.0

^† ^Summary statistics are at the individual student level.

School lunch patterns and vending machines were examined across gender and grade level. Boys ate meals from the main lunch line more frequently than girls (M = 2.8 ± 2.0 vs. 2.0 ± 1.8 days/week; p < .001) and brought lunch from home less frequently than girls (M = 0.7 ± 1.6 vs. 1.1 ± 1.8 days/week, p < .001). Boys purchased soft drinks from vending machines on more days/week than girls (M = 1.8 ± 1.8 vs. 1.4 ± 1.6; p < .001). There were no gender differences in a la carte, fast food, and convenience store lunch purchases or in snack food vending machine purchases across gender (data not shown).

A la carte, fast food restaurant, and convenience store lunch practices differed across grade in school. A la carte food purchases were most frequent among students in the 9^th ^and 10^th ^grades and declined among students in the 11^th ^and 12^th ^grades (M = 2.0 ± 1.8 2.1 ± 1.7, 1.7 ± 1.6, and 1.5 ± 1.6 days/week in 9^th^, 10^th^, 11^th^, and 12^th ^graders, respectively; p < .001). In contrast, eating lunch at a fast food restaurant was much more frequent among youth in 11^th ^and 12^th ^grades (M = 0.1 ± 0.6, 0.2 ± 0.6, 0.4 ± 1.0, and 0.7 ± 1.3 days/week in 9^th^, 10^th^, 11^th^, and 12^th ^graders, respectively; p < .001). Getting lunch from a convenience store was not common, but youth in upper grades reported getting lunch more frequently from convenience stores than youth in lower grades (M = 0.1 ± 0.5, 0.1 ± 0.5, 0.2 ± 0.5, and 0.3 ± 0.8 days/week in 9^th^, 10^th^, 11^th^, and 12^th ^graders, respectively; p < .001). Eating from the regular lunch line, bringing lunch from home, and snack and soft drink vending machine purchases did not differ across grade in school (data not shown).

### School food environment and policies

Variables assessing the school food environment and policies of potential relevance to students' lunch patterns and vending machine practices were examined (Table 2). About two-thirds of the schools had a closed campus policy during lunchtime (68.4% n = 13 schools). With regard to vending machines, only three (15.8%) of the schools had policies regarding the types of food that could be sold in vending machines. The mean number of snack vending machines in each school was 2.7; four schools had none, six schools had 1–2, six schools had 3–4, and four schools had 5–7 snack vending machines. The mean number of soft drink vending machines in each school was 5.3; three schools had 1–2, seven schools had 3–4, six schools had 5–7, and four schools had 8+ soft drink vending machines. Out of the 16 schools that had snack vending machines, 25% (n = 4), had them closed during lunchtime. Out of the 20 schools that had soft drink machines, 55% (n = 11) had them closed during lunchtime.

**Table 2:** School food policies and environment in 20 high schools

School policies:	Yes	No	Percent with policy
Closed campus policy during lunch	13^†^	6^†^	68%
Policies about food sold in vending machines	3^†^	16^†^	16%
Vending machines closed during lunch			
Snack machines	4	12^‡^	25%
Soft-drink machines	11	9	55%
			
Number of vending machines/school:	Mean	SD	
Snack Machines	2.7	2.5	
Soft-drink machines	5.3	2.7	
Other type of machine offerings	5.1	2.4	

^† ^One (1) school missing

^‡ ^Four (4) schools had no snack vending machines

### Open/closed campus policy during lunchtime and student lunch practices

As shown in Table 3, students at schools with open campus policies during lunchtime were significantly more likely to eat lunch at a fast food restaurant (0.7 days/week vs. 0.2 days/week) or a convenience store (0.3 days/week vs. 0.1 days/week) than students at schools with closed campus policies. There were no significant differences for eating from the main lunch line, eating a la carte foods, or bringing lunch from home between students at school with open campus policies vs. closed campus policies.

**Table 3:** Student lunch patterns (days/week) by school policies regarding open/closed policy during lunch hours

	Closed campus (N = 13 schools)	Open campus (N = 6 schools)	
	Mean (SD)	Mean (SD)	p-value

Regular lunch line	2.5 (2.0)	2.0 (1.8)	.156
Ala carte lunch	1.8 (1.7)	1.6 (1.6)	.493
Bring lunch	0.9 (1.7)	1.1 (1.8)	.344
Fast food restaurant	0.2 (0.8)	0.7 (1.3)	<.001
Convenience store	0.1 (0.4)	0.3 (0.8)	<.001

### School policies/availability of vending machines and student vending machine practices

Having a school policy about the types of foods sold in vending machines was significantly inversely associated with frequency of student snack food purchases from vending machines (Table 4). In schools with policies, students reported making snack food purchases an average of 0.5 days/week as compared to an average of 0.9 days/week in schools without policies. Similar nonsignificant trends were found for soft drink purchases.

**Table 4:** Student vending machine (VM) practices by policies regarding food sold in VM, policies regarding hours of operation of VM, and number of VM at school

	Policy about food sold in VM			VM closed during lunch^†^			Number of VM^†^					
	Yes	No		Yes	No		0	1–2	3–4	6+	8+	

	M (SD)	M (SD)	p-value	M (SD)	M (SD)	p-value	M (SD)	M (SD)	M (SD)	M (SD)	M (SD)	p-value

Student snack food VM purchases (days/week)	0.5 (1.1)	0.9 (1.3)	<.001	0.9 (1.2)	1.0 (1.3)	.477	0.4 (1.0)	0.8 (1.2)	1.1 (1.4)	1.1 (1.4)		<.001
Student soft drink VM purchases (days/week)	1.4 (1.7)	1.6 (1.7)	.110	1.4 (1.6)	1.9 (1.8)	.043		1.3 (1.6)	1.7 (1.7)	1.4 (1.6)	1.9 (1.8)	.173

^† ^Done for policies regarding school snack and soft drink policies in accordance with whether student or soft drink

VM purchases are being examined.

Associations between vending machine availability at schools and student snack food and soft drink vending purchases were also examined (Table 4). Student snack food purchases from vending machines were significantly more frequent among students from schools with a greater number of snack food vending machines. Policies regarding hours of operation of snack food machines were not associated with snack food purchases in the 16 schools that had snack machines. In contrast, student soft drink purchases from vending machines were not significantly associated with the number of soft drink vending machines, but were significantly lower in schools in which machines were turned off during lunchtime.

## Discussion

This study examined associations between school food policies and student lunch practices and vending machine purchases. Study findings have implications for schools and suggest steps that schools could take to encourage healthier eating practices among students. A closed campus policy during the lunch hour was associated with fewer lunch purchases from fast food restaurants and convenience stores by students. The existence of school policies regarding the types of foods that can be sold in vending machines was associated with fewer student snack food purchases from vending machines. Student snack food purchases from vending machines was also associated with the number of snack food vending machines at school. Finally, limited hours of operation of soft drink vending machines was associated with fewer student purchases of soft drinks from vending machines. While previous studies have found associations between the food school environment (e.g, number of vending machines, availability of a la carte foods) and student eating behaviors [19,20], to the best of our knowledge, this is the first study that has examined and observed associations between school food policies and student eating behaviors.

It is encouraging to note that students most frequently reported eating the regular school lunch, which is regulated in terms of nutritional standards. However, the students also reported frequent consumption of a la carte foods for lunch, which have only minimal regulations in terms of nutrition. Previous analyses have shown that the most commonly available a la carte foods within schools tend to be high in energy and low in nutrients [22,25,26]. The high intake of a la carte foods points to a need for ensuring that healthy foods that are appealing to high school students and reasonably priced, are offered as a la carte choices, and that access to food high in fat and sugar is limited. The finding that high school students bring lunch from home an average of about once a week suggests that interventions aimed at improving the dietary intake of adolescents might also include ideas for healthy brown bag alternatives.

Differences in school lunch and vending machine practices across grade in school suggest that as students enter the upper grades, they are less likely to eat a la carte foods within the school and are more likely to make food purchases outside the school premises at fast food restaurants and convenience stores. These findings suggest that avenues for "alternative" and probably less healthful and more costly food options change as youth get older and have more independence due to having cars and being allowed the freedom to leave school during the day. School-based interventions need to take into account the different eating patterns of younger and older students. Interventions also need take consider factors likely to be influencing school eating practices such as the proximity of different food outlets to the school campus.

Strengths and limitations of the study need to be taken into account in interpreting the findings. A major strength of this study, which contributes to the utility of the findings, was that data on food policies and the school food environment were collected from multiple sources including principals, food service directors, and observations by trained research staff. Since data were collected from 20 schools, which differed from each other in terms of school size, populations served, and school food environments and some generalizations of the findings are possible. However, the 20 schools that participated in the study were from one Midwestern state in the United States and served a population with low representation of minorities and adolescents from lower socio-economic levels. Thus, caution should be taken in making generalizations to other areas and populations. The wording of questions on the student survey also limited some of the conclusions that we were able to draw from the data. For example, for vending machine purchases, questions asked about days per week that students made purchases. The item did not assess number of items purchased per day so could not capture students making numerous purchases in a single day. Finally, additional questions on student eating practices (e.g., fast food intake) after school hours and total dietary intake would have also been informative.

Further research is needed to explore the impact of changing the school food environment and policies on student eating practices both during, and after, school hours. For example, if schools implement closed campus policies during lunchtime, will students make healthier food choices at school or eat more of the less healthful a la carte choices? And will they eat more or less frequently at fast food restaurants after school? If vending machines in schools offered fewer high fat and high sugar foods and more healthful options, what would students choose to drink at school and after school? Further research is also needed to replicate findings from this study in different school populations and assess different types of food policies.

## Conclusion

In conclusion, school food policies that decrease access to foods high in fats and sugars are associated with less frequent purchase of these items among high school students. Based upon these findings, it is recommended that schools examine their food-related policies and consider policies to decrease access to foods and beverages that are low in nutrients and high in fats and sugars. Strategies suggested by our data include having closed campus policies during the lunch hour, having policies regarding the types of food that can be sold in vending machines (e.g., more healthful options), keeping soft drink machines turned off during the lunch hour, and limiting the number of snack food vending machines. Schools should also consider strategies for making healthier alternatives more accessible and attractive to students in terms of appearance, taste, and cost. Clearly factors other than eating practices at school are associated with the overall quality of dietary intake and health outcomes of youth; nevertheless, since 35–40% of calories are consumed at school [23,24], eating practices at school are likely to be making a significant contribution. As educational institutions, schools have a crucial role to play in providing youth with healthy eating opportunities.

## Competing interests

The author(s) declare that they have no competing interests.

## Authors' contributions

DNS wrote the manuscript and worked with the analyst to develop an analysis plan and incorporated input from all other authors on the manuscript. SAF developed and directed the overall study. PJH contributed to the study design and conducted the data analysis. MS contributed to the study design and implementation. JAF contributed to study implementation and oversaw evaluation activities.
